# Synthesis and characterization of aramid composites reinforced with silanized graphene platelets†

**DOI:** 10.1039/d2ra04797g

**Published:** 2022-09-21

**Authors:** Abdullah Alhendal, Jessy Shiju, Mohamed Rashad, Fakhreia Al-Sagheer, Zahoor Ahmad

**Affiliations:** Department of Chemistry, Kuwait University P. O. Box 5969 Safat 13060 Kuwait abdullah.alhendal@ku.edu.kw

## Abstract

The synthesis and characterization of aramid composites reinforced with graphene platelets are reported. Hydroxy-functionalised graphene platelets were modified with two sol–gel binders (aminopropyl- or aminophenyl-trialkoxysilanes) and then chemically linked with aramid chains. The effect of the two sol–gel binders on the physiochemical and mechanical properties was evaluated. Chemical changes during the sol–gel reaction and subsequent amidation process in the nano-composite preparation were evaluated by the XPS and FTIR analyses. Thin films of these composites with different proportions of graphene were prepared. Morphology of the hybrids prepared was studied by the SEM technique. Properties of the composite films were studied by dynamical mechanical thermal (DMT) analysis to measure their glass transition temperature (*T*_g_) and storage modulus. These properties have been compared with previously reported values using pristine graphene (Gr) as a filler. The increase in thermal mechanical properties on addition of silanized graphene (SiGr) showed a large shift in the *T*_g_ and more increase in storage modulus by chemically binding SiGr sheets on the aramid chains. Aminophenyl-trialkoxysilane was found to give better results due to the presence of phenyl groups which were more rigid than propyl groups present in aminopropyl-trialkoxysilane. The effect of chemical bonding and the possible π–π secondary bond interactions between the matrix and graphene platelets on the properties of the resulting hybrids are discussed.

## Abstract 2

The synthesis and characterization of aramid composites reinforced with graphene platelets are reported.

## Graphical abstract

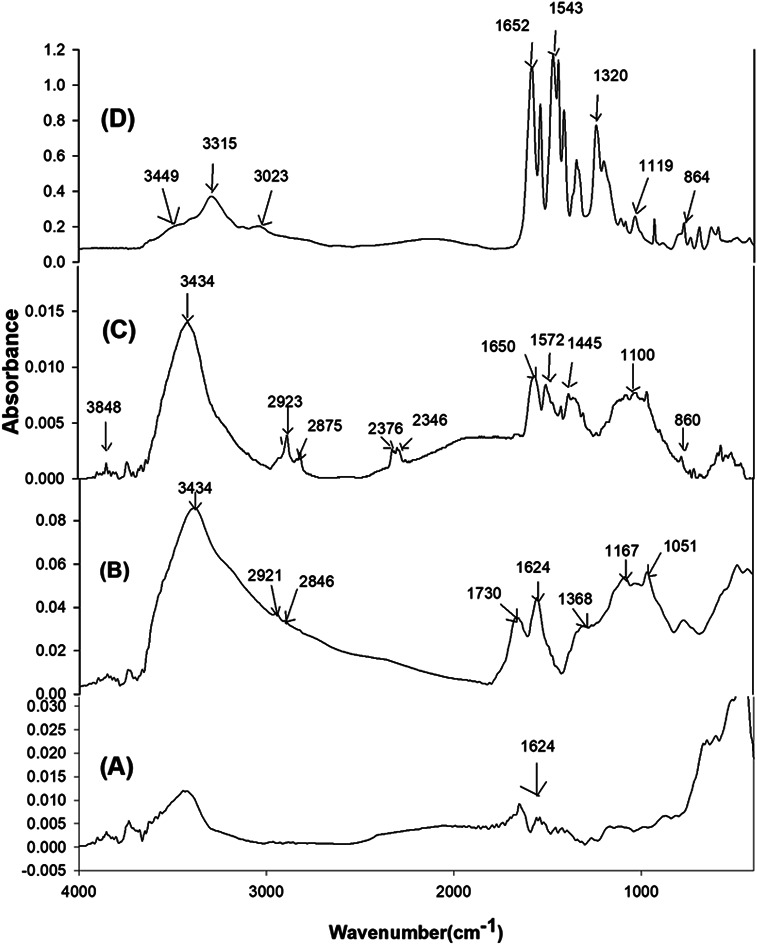

## Introduction

The last few decades have witnessed a rapid development in the field of polymer composites^1–4^ mainly for the reason that pure polymeric materials, in general, were unable to meet the specific high-performance requirements of the modern technological applications. Polymer composites can provide high mechanical strength and thermal stability with diverse properties which are substantially different or even better as required for the advanced applications. This is possible, in particular, when the filler is present at the nanoscale^5–7^ and is distributed homogenously in the matrix. Due to the unique and tunable properties of network structures, the use of inorganic materials in combination with high performance polymers has attracted a lot of interest in the recent past for numerous applications in the aerospace and electronics industries.^8–10^ A good number of the publications have been focussed on nano-inorganic particles produced by *in situ* sol–gel processes^11,12^ in the matrix or as surface coating materials. The layered silicate structures^13–15^ using different types of clays also became very popular to enhance the toughness of the polymeric materials. Carbon-based nano-fillers in the recent times, however, are being preferred in comparison to clay or other inorganic metal oxides to improve the mechanical strength of polymers. Polymer composites using carbon black^16,17^ carbon nanotubes (CNTs)^18–24^ carbon fibre^25,26^ and graphene (Gr)^27–29^ have shown a large improvement in thermal, mechanical, conductive and flame-retardant properties.

Among the carbon materials, CNTs have proven to be very effective as conductive fillers and a wide variety of CNT–polymer composites using different techniques have been reported.^18–24,30,31^ Chemical functionalization of CNTs can increase their interaction with the polymeric matrix facilitating their processability.^21,32^ The presence of a suitable solvent can greatly enhance their de-aggregation required for a uniform dispersion^33^ in the matrix. The present authors have shown that strong interfacial interactions of the matrix with the surface-modified CNTs reduced the stress-transfer problem in the composite material and resulted in higher modulus of 4.26 GPa and a glass transition temperature of 338.5 °C, whereas the CTE was reduced to 101.8 ppm on addition of only 2.5 wt% CNTs in aramid matrix^24,35^. Because of their high aspect ratio, the alignment of the CNTs in a matrix can create a large polymer–filler interface thus producing large reinforcement. But one of the major disadvantages with CNTs is their entanglement tendencies due to which the increased viscosity with higher loadings may lead to a significantly poorer dispersion.^34^ The alignment of CNTs, even at a laboratory scale^35^ seems difficult to achieve at present. Another disadvantage with CNTs is their higher cost of production which may discourage their uses for production of such composites on the industrial scale.^31,36^ Graphene on the other hand is cheaper in comparison to CNTs with exceptionally good mechanical, thermal and electrical properties. Owing to their high surface area, large aspect ratio and high tensile strength and thermal conductivity these can be very suitable to improve the properties of polymeric matrices.^27,37,38^ It is also much easier to process these platelets at higher loadings in the matrix, as the viscosity of the polymer–graphene mixture is significantly lower than the CNT composites. These platelets due to their micron-size wrinkled layered structure have been found to be a highly efficient load bearer filler in comparison to the CNTs for the polymer matrices.^39^ The improvement in the ultimate physicochemical characteristics of such composites, however, depends on the distribution of Gr layers in the polymer matrix as well as on the interfacial bonding between the Gr and the matrix.^40^ Modification of the Gr surface is usually carried out by oxidation followed by its chemical functionalization.^41^ The oxidized graphene (GrO) bearing various polar *i.e.*, hydroxyl and carboxyl functional groups can alter the van der Waals interactions significantly. These methods make Gr more compatible with organic polymers.^42^ The covalent or the noncovalent functionalization facilitate dispersion and stabilize Gr in the matrix by preventing its agglomeration.^43–45^ For this reason, GrO is now being used extensively in industry as a nanofiller in the polymer composites.^46,47^

The GrO surface can be further modified by the sol–gel process using different types of silanes. The silica network structure can help to increase the interlayer distance thus reducing the agglomeration tendencies of the Gr platelets. Jing Li *et al.*^48^ modified GrO surfaces using tetraethoxysilane and the silanized graphene (SiGr) was found to improve the protective properties of composite coatings. High performance benzoxazine–bismaleimide resin based SiGr composites were prepared by Jiang *et al.*^49^ who showed that SiGr has good dispersity in the matrix. Thermal decomposition temperature and char yield of the composites were reported to improve markedly. Organically modified silanes represented by M–Si–(OR)_3_ can be used through the sol–gel processing to link the oxidized graphene with polymer chain. The trialkoxy (OR) can be easily hydrolysed and condensed using sol–gel process and linked with hydroxy functionalized Gr. Depending on the nature of the functional group M, the silanized graphene then can be linked to a polymer chain. The silica network produced during the silanization process can greatly increase the interlayer distance and creates a disorder in the Gr lattice thus ensuring a uniform distribution in the matrix. The GrO was modified by Liu *et al.*^50^ using γ-glycidoxypropyltrimethoxysilane to prepare the polyurethane composites. The SiGr provided a good compatibility with the matrix thus increasing the thermal decomposition temperature of polyurethane matrix.

Aromatic polyamides^51^ due to their high specific strength and thermal stability with a lower density are considered as very important commercial fibers. In the present work thermal mechanical properties of such easily processable aromatic polyamide chains were improved by reinforcement with SiGr using two different types of organically modified silanes *i.e.*, aminophenyltrimethylsilane APhTMS and aminopropyltriethoxysilane APrTES. To improve the solubility (processability) of poly(*p*-phenylene-terephthalamide) chains, generally known as Kevlar© in industry, they were modified by introducing an optimum number of *meta*-linkages in the chains. The sol–gel process was used to bond the aminosilanes on the surface of hydroxy functionalised graphene, which then acted as a link between the graphene platelets and the polymer matrix chain. Viscoelastic properties of these chemically bonded hybrids with different proportions of SiGr have been determined and compared with our earlier work with those hybrids, which were reinforced with the pristine Gr.^43^

## Experimental section

### Chemicals and materials

The monomers terephthaloyl chloride (99%), *para*- and *meta*-phenylenediamines (99%) and aminopropyltriethoxysilane (98%) (APrTES), HCl and the solvent dimethyl acetamide (DMAc) with water contents less than 0.004%, all of analytical reagents (AR) grades were procured from Sigma-Aldrich. Graphene oxide powder (99.4%), (plate diameter *Ø*: 1.5–5.5 μm and thickness: 0.43–1.23 nm) was obtained from Nano US Research Nano materials. 3-Aminophenyltrimethoxysilane (90%) (APhTMS) was obtained from Gelest.

### Preparation of aramid graphene composites

#### Aramid resin

A mixture of *meta*- and *para*-phenylenediamines taken respectively in the molar ratio 35 : 65 was placed in a 300 ml conical flask in a glove box under complete anhydrous conditions. The total moles of amines taken were 0.025 and, in this mixture, 110 g of DMAc as solvent was added. The mixture was stirred with magnetic stirrer for 30 min until dissolved completely. Under complete anhydrous condition terephthaloyl chloride (0.025 mol) was then added to react with phenylenediamines. Stirring of the reaction mixture continued for the 24 h to complete the polymerization reaction. The –COCl terminated aramid matrix chains were prepared by further addition of 2.8 millimole of terephthaloyl chloride in the reaction mixture. Further stirring continued for 3 h to complete the endcapping of the chains with –COCl groups.

### Propyl- and phenyl-substituted silanized graphene

**Scheme 1:**
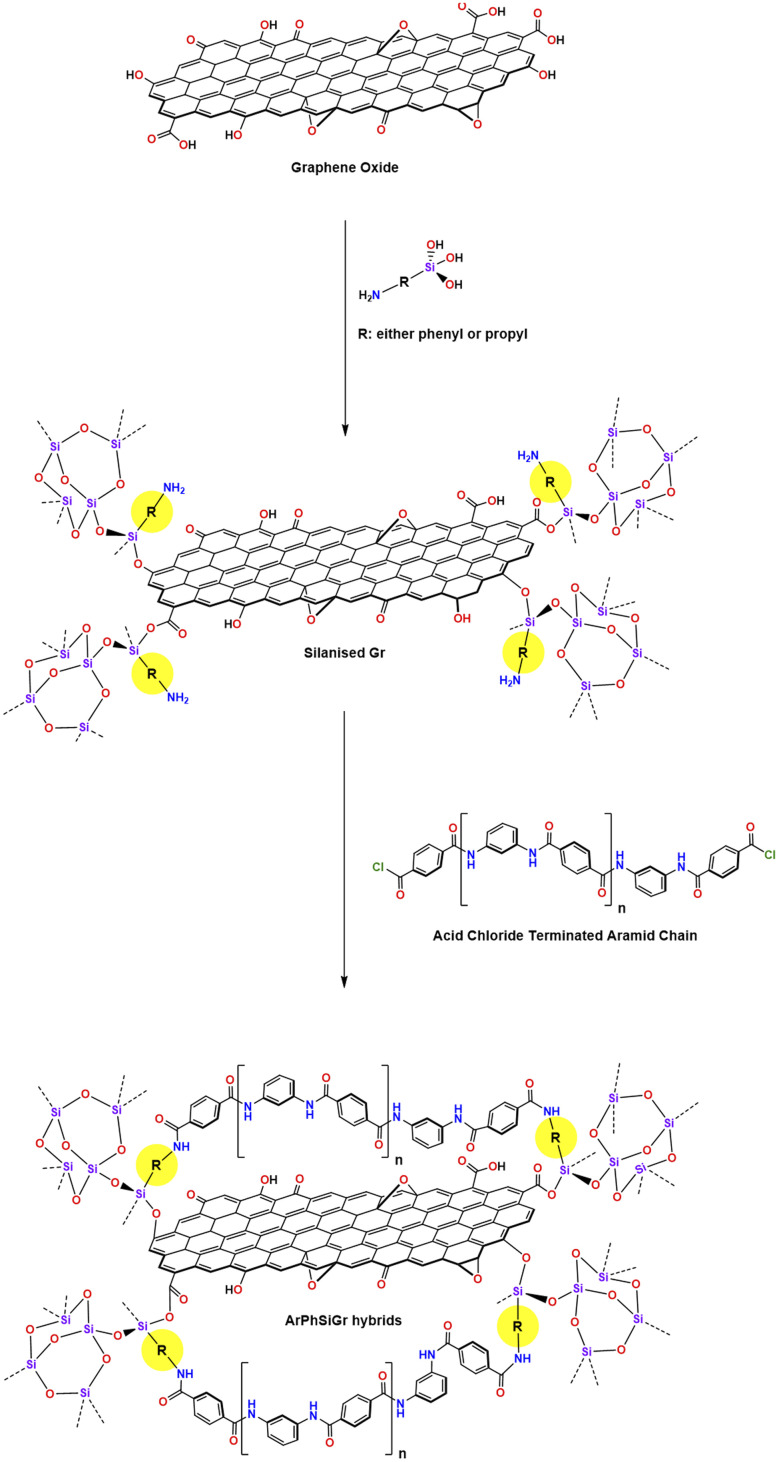


The oxidized graphene (0.30 g) was taken in 20 g DMAc in a 150 ml flask and stirred for 24 h then followed by 2 h sonification. The sol–gel process was then carried out to functionalize the surface of graphene with aminosilanes. Two different types of silanes *i.e.*, APhTMS and APrTES were used in this work (herein, the described method is identical for two composites) and 2.8 millimole of each silane was separately added to the graphene solution and the mixture was stirred for 30 min for complete mixing. To this solution, water was added in the form of DMAc solution (taken in 5 : 95 ratio respectively by weight) to carry out the sol–gel reaction. The ratio of silane to water was kept respectively at 1 : 1.5. Few drops of HCl (0.05 M) were added to catalyze the sol–gel reaction. The mixture was continuously stirred at 70 °C for 4 h on a magnetic hotplate. The stirring further continued at room temperature for 24 h. The silanized graphene solution was used as stock solution to prepare (Scheme 1) the silanized graphene hybrids with the aramid resin.

### Aramid-silanized graphene hybrid films

Different amounts of two silanized graphene solutions were added separately to a known amount of ClOC-endcapped polymer solution with stirring continued for another 24 h to get the complete mixing (Scheme 1). Thin hybrid films containing different amounts of SiGr were cast in glass Petri dishes by evaporating the DMAC at 80 °C. These films were then soaked in distilled water to leach out any HCl produced during the reaction of terephthaloyl chloride with phenylenediamines. Afterwards, the films were dried at RT for three days and then at 110 °C under vacuum for four days. The hybrid films prepared from the silanized graphene using APhTMS are referred as ArPhSiGr and those prepared from APrTES are referred as ArPrSiGr where the last digit denotes the wt% of the filler SiGr (silanized graphene) in the aramid matrix.

### Characterization of the hybrid films

The absorption spectra were obtained for the pristine and oxidized Gr, SiGr, ArPhSiGr and ArPrSiGr hybrid films in the range of 400–4000 cm^−1^ using the JASCO FTIR Spectrometer-6300 to monitor the silanization procedure. The composition of different elements *i.e.*, carbon, oxygen, nitrogen and silicon present on the film surface were determined by XPS to identify the functional groups present during the preparation of the hybrid materials. Thermo ESCALAB 250 Xi with a monochromatic radiation source was used for this purpose. FESEM was using to study the morphology of the composite films. The films fractured using the microtome at very low temperature and coated with gold were examined at 15 kV using SEM User, Interface with Model: SUPRA 50 VP(JDISS). The visco-elastic properties of the hybrid films were measured on Dynamic Mechanical Analyzer Q-800 (TA instruments). The storage modulus of the hybrid films at different loadings of SiGr were measured as function of temperature. The glass transition temperature was measured from the peak of tan *δ* plot *vs.* temperature. The measurements were taken under tension mode in the temperature range 50–500 °C, at the heating rate of 5 min °C^−1^ using frequency 5 Hz under nitrogen gas at the floating pressure of 60 Pa. Thermogravimetry was performed on approximately 10 mg of the sample from ambient to 800 °C at a heating rate of 10 °C min^−1^ in a dynamic nitrogen atmosphere (50 ml min^−1^), using TGA-50 TA automatic analyzer.

## Results and discussion

### Characterization of the polymeric composite

**Fig. 1:**
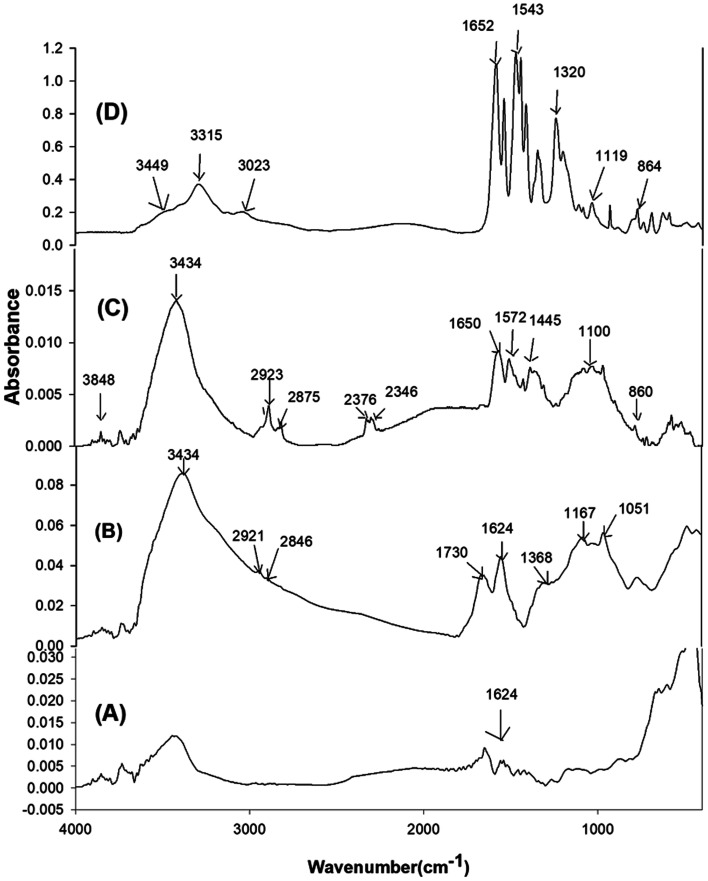
FTIR spectra: Gr (A). GrO (B), SiGr (C), ArPhSiGr-8 hybrid (D).

**Fig. 2:**
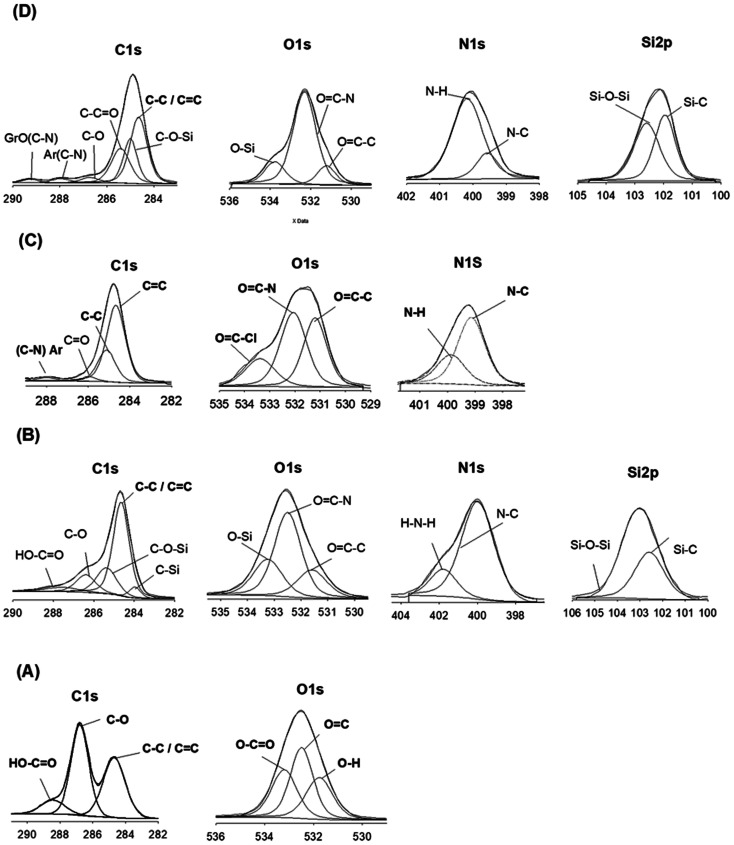
XPS spectra: GrO (A), SiGr (B), Ar neat (C), ArPhSiGr-8 hybrid (D).

FTIR spectra of the pristine Gr, GrO, SiGr and the ArPhSiGr hybrid film are given in Fig. 1 to confirm the modification of Gr surface and chemical bonding between the aramid chain and Gr surface (a comparable FTIR study for the ArPrSGr is provided in the ESI†). The peak at 1624 cm^−1^ for (C=C) bonds in spectrum (A) shows the presence of the hexagonal structure of pristine Gr whereas the peaks significant in spectrum (B) for GrO correspond to the functionalities created by the oxidation of Gr. A broad absorption peak between the 2600 cm^−1^ and 3500 cm^−1^ is mainly related to the hydroxyl groups on the GrO that were used in the present work for the sol–gel reaction. Two small peaks at 2921 cm^−1^ and 2846 cm^−1^ can be attributed to the (C–H) asymmetric and symmetric stretching vibrations derived from long alkyl chain of Gr. The adsorption band at 1730 cm^−1^ and 1625.6 cm^−1^ correspond to the C=O stretching (from carbonyl and carboxyl groups) and aromatic C=C vibration that results from skeletal vibration from unoxidized graphitic domains respectively. Besides, two weak peaks present at 1167 cm^−1^ and at 1051 cm^−1^ are from the C–O stretching deformation mode of the carboxylic acid group and epoxide functional group respectively. The peak at 1368 cm^−1^ can be attributed to single bonded carboxyl group (C–O)–O. The high intensity of the peaks due to oxygen functional groups is useful to develop further links of GrO platelets surface with silica by the sol–gel processing and then through amine groups with the matrix chain (Fig. 2).

Spectrum (C) in Fig. 1 represent the SiGr obtained after carrying out the sol–gel reaction using APhTMS with Gr. A drastic decrease in the hydroxyl group peaks can be attributed to their reaction with the alkoxy groups of the APhTMS. The peaks at 1563 cm^−1^ and 1653 cm^−1^ are attributed to the C–N stretching and N–H bending of the unreacted amine group of the silane. The absorption in the range 1000–1150 cm^−1^ is for Si–O–Si network (asymmetric stretching).^52^ The new bands at 1116 cm^−1^ and 1034 cm^−1^ are, therefore, assigned to the Si–O–Si network. The study conducted by Musto *et al.*^53^ has shown that Si–O–Si absorption at higher wave numbers represent the cyclic whereas at the lower wave number the linear silica structure. The absorption at 914 cm^−1^ is due to Si–OH bonds. The presence of APhTMS in the spectrum (C) is also confirmed by presence of the Si–C bond appearing at 720 cm^−1^.^54^ The decrease of broad peak of carboxylic –OH and C–OH around 3000–3500 cm^−1^ means that the amount of silane used was sufficient to cover the majority of the surface of graphene.

The spectrum (D) in the Fig. 1 represents the hybrid film ArPhSiGr-8. The peaks at 1652 cm^−1^, 1543 cm^−1^ and 1320 cm^−1^ are attributed to amide functional groups which are from aramid and from the amidation reaction between the amine group of APhTMS and –COCl groups of aramid whereas the peak at 3315 cm^−1^ is due to –NH stretching. The new composite bands at 1119 cm^−1^ and higher wavenumber and at 864 cm^−1^ are assigned to the and Si–O–Si and Si–OH respectively. A small peak at 3449 cm^−1^ seems due to OH groups from the Si–OH produced during sol–gel reaction. The FTIR results shown in the spectra (C) and (D) approves the silanization process. The results obtained for the silanization process and the hybrids using APrTES were of similar nature, and these are not presented here to avoid the repetition chemical binding between the graphene and amide chain has further been investigated by the XPS analysis.

XPS analysis was employed to elucidate the surface composition of Gr, GrO, SiGr, aramid and the hybrid films to provide more information on the interactions between silane coupling agent and the bonding between SiGr and aramid matrix chains. In the XPS spectrum (A) obtained for GrO, the peaks at 284.68 eV, 286.79 eV and 288.43 eV are attributed to the sp^2^ carbon bonds, C–O bonds, C=O bonds respectively. In the spectrum (A) as expected N 1s peak is absent.

The spectrum (B) shows the presence of both Si and N elements in the SiGr and the concomitant reduction of carbon atoms with respect to GrO that confirm the silane functionalization by APhTMS through the reaction of the alkoxy group of the silane with –OH groups of GrO. Additional peaks at 283.97 eV and 285.37 can be assigned to C bonded to Si owing to the reaction with silane coupling agent. The atomic wt% of carbonyl carbon structure in the SiGr in comparison to GrO decreased considerably *i.e.*, from 29.43% to 7.96%. The peak from unreacted –NH_2_ groups of APhTMS is still present at 401.8 eV which indicates the silanization reaction occurred through alkoxy groups of APhTMS and the NH_2_ groups are retained.

The XPS spectrum (C) for the aramid chains end-capped with –COCl groups shows peaks at 284.67 eV for C–C as well as 285.11 eV for the C=C bond. The (C–N) bond formed in the aramid chain or (C–N)–Ar appears at 287.90 eV. The unreacted COCl on aramid chains are confirmed in the carbon spectrum at 289.08 eV. Oxygen peaks from O=C–C appears at 531.2 whereas O=C–N can be seen at 532.06 eV. The unreacted terminal acid chloride peak O=C–Cl is seen at 533.38 eV and N–C and N–H peaks can be at seen at 399.51 eV and at 400.05 eV. In spectrum (D) for ArPhSiGr-8 hybrid film the disappearance of COCl at 289.08 eV and O=C–Cl at 533.38 eV and that of –NH_2_ peak at 401.79 eV confirms the reaction taking place between the acid chloride terminated aramid resin and silanized Gr. The atomic wt% of O=C–N in the composite increased as well due to the amide bond formation. The peak of the binding energy (BE) for a specific element depends on the structure and local chemical environment of that element. The O=C–C peaks from GrO and silanized Gr are at 532.5 eV and that from neat aramid and its hybrids at 531.2 eV and 531.23 eV respectively. The BE values in the hybrid film are closer to aramid since the matrix contributed more than 92 wt% of the hybrid system.

### Composite's morphology

**Fig. 3:**
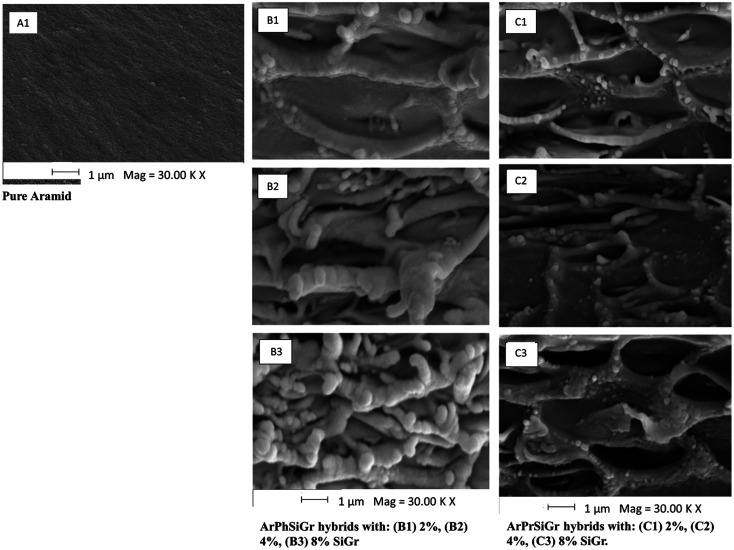
SEM images of neat Ar (A), ArPhSiGr (B) and ArPrSiGr (C) hybrids with: 2%, 4%, 8% SiGr.

Scanning electron microscopy imaging was used to evaluate the surface morphology and graphene distribution in the matrix of the composite films produced from two different aminosilanes. Fig. 3A1 show the typical morphology of pure aramid polymer while hybrid nanocomposite at three different loadings of SiGr; ArPhSiGr and ArPrSiGr are illustrated in Fig. 3B and C, respectively. The micrograph images show that silanization have promoted the unfolding with the formation of well-exfoliated graphene sheets. There are no signs of restacking of graphene nano-sheets which are uniformly dispersed in the aramid matrix. Due to the fact that GrO platelets are more prone to lateral cleavage, we find a marginal decrease in the lateral side of the platelets. The increase in the thickness of graphene platelets is due to the presence of silica network on the surface of Gr which further have been linked with the polymer chains. There are no visible signs of de-bonding or pull-out of Gr sheets from the matrix chains in the micrographs. A loose network structure in the matrix can be seen where the Gr sheets have been linked through chemical bonds with the polymer chains, in fact the polymer chains got imbibed within the silanized surface of the Gr sheets. The increased loading of Grs has drastically increased the irregular interphase protuberances strongly bonded and imbibed with the matrix chains. The wrinkling effect on the 2D graphene nano-sheets is also evident which has increased the contact with the polymer chains, and it can further increase the toughness of the hybrid material.

Comparing the morphology of the films obtained using APhTMS (Fig. 3B) with those of APrTES (Fig. 3C) as coupling agents, silanized Gr sheets are puffed-up which can be attributed to more interactions between the filler and the matrix in the former case. This is due to presence of phenyl groups in case of ArPhSiGr hybrids. The π–π interactions can take place between the free electrons of phenyl groups on silanized Gr and the extended phenyl groups of the matrix chain thus developing more secondary bonding interactions between the filler and the matrix. The improved dispersion and imbibition of polymer chain on the surface of Gr as seen in Fig. 3B and C is due to both primary and secondary bonding created by aminosilane on the surface of graphene and its linkage with the matrix chains, which can have a positive effect on the ultimate strength of the composite material.

### Thermal mechanical properties

**Fig. 4:**
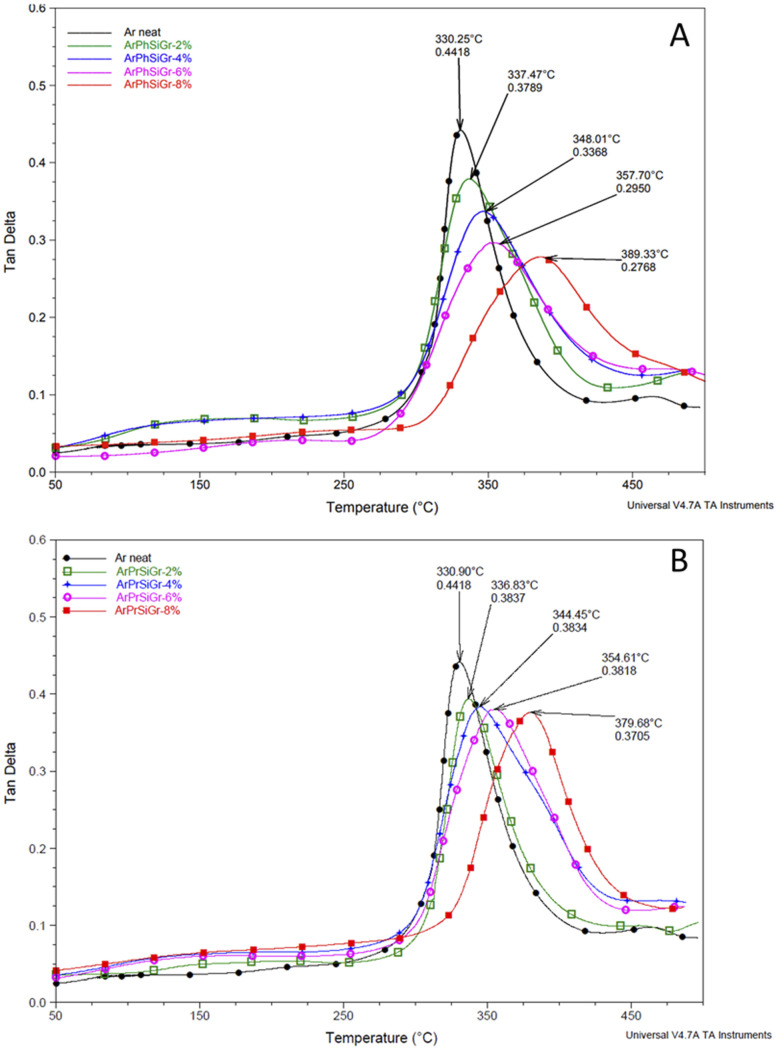
Temperature variation of tan *d* in ArPhSiGr hybrids (A), ArPrSiGr hybrids (B); SiGr wt%: 0 (●), 2% (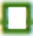), 4% (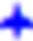), 6% (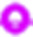) and 8% (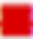).

Thermal mechanical properties of aramid-silanized graphene hybrid films were studied by measuring the stress response during the exposure of the film to a sinusoidal strain within the temperature range of 25 to 500 °C. The results were obtained in terms of the storage modulus (*E*′) and tangent psi (tan *δ*) as a function of the temperature. The temperature dependence of tan *δ* can reveal different relaxation processes in the material denoted by α, β and γ transitions. Fig. 4 shows the effect of temperature on the tan *δ* curve for ArPhSiGr (Fig. 4A) and ArPrSiGr (Fig. 4B) composites with different wt% of SiGr. With increase in temperature, the onset of segmental motion led to a sharp increase at 330 °C in tan *δ* curve for neat aramid and this peak corresponds to the α-relaxation temperature (*T*_g_). With increase in the loading of silanized graphene the maxima of the curve shifts to higher temperature in both types of hybrids indicating that the reduced mobility of the polymer chains. The surface of graphene is covered with porous silica network and the polymer chains are not only linked with it chemically, but the chains were imbibed within this network as seen in the SEM images. The strong adhesion of the polymer chains with SiGr is also confirmed from the dampening of the tan *d* curves, which increases as the elastic nature of the material increases as SiGr is increased in the matrix. The *T*_g_ for the pure polymer increased from 330 °C to 379 °C for the ArPrSiGr system and to 389 °C for the ArPhSiGr system with 8% SiGr. The increment in *T*_g_ is attributed to the suppressed mobility of aramid back bone due to the increased interfacial interaction and the chemical bonding of the polymer chain and on the surface of Gr. This can be attributed to the presence of silica network produced by the sol–gel process has separated the sheets apart thus increasing its free surface to interact with polymer matrix. Chemical bonding between the amine groups of the silanized graphene and the acid chloride end-groups from aramid matrix has further improved their adhesion with the matrix resulting an increase in the *T*_g_ of the hybrid material.

**Fig. 5:**
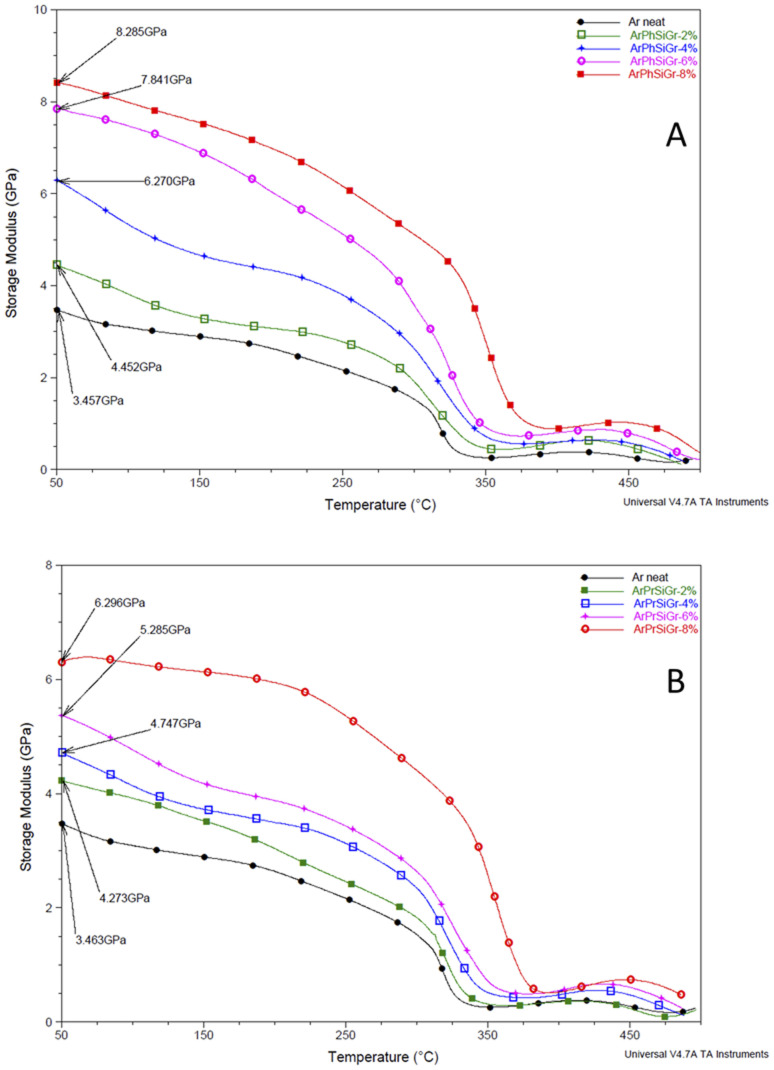
Temperature variation of storage modulus (GPa) in ArPhSiGr (A) and ArPrSiGr (B) composites; SiGr wt%: 0 (●), 2% (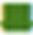), 4% (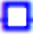), 6% (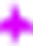) and 8% (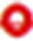).

Liao *et al.*,^55^ in their extensive review on the study of variation of *T*_g_ in the graphene composites with different polymers has reported that increase in the glass transition temperature from the neat polymer has been, in general, very nominal. Several studies showed no increase in *T*_g_ when the preparation techniques like melt blending were used. The pristine Gr in fact is full of conjugated C=C which are too stable to provide interaction in particular with the aliphatic type of polymer backbones. But in our case, we have used aromatic polyamide which has lot of phenyl groups on the chain. In Fig. 5 we compare our previous work using pristine Gr^45^ with that of two types of hybrids using aminosilanes. The mobile pi electrons (π and π*), present on both sides of the Gr sheets can have multiple π–π interactions with phenyl rings of aramid matrix we used. Even using pristine Gr, therefore, we found an increase in the *T*_g_ on loading with pristine Gr. A comparison in Fig. S2† shows that the silica network produced from aminosilanes during the sol–gel process was immensely helpful in keeping the nanosheets apart thus creating more surface area for chemical bonding between Gr and the matrix. A much higher increase in *T*_g_ is, therefore, observed when SiGr used as fillers (Table 1).

**Table 1:** DMTA comparison of Ar–Gr/ArPrSiGr/ArPhSiGr composites

Sample	Storage modulus (GPa)			*T*_g_ (°C)		
	ArGr	ArPrSiGr	ArPhSiGr	ArGr	ArPrSiGr	ArPhSiGr
Ar–Gr-0%	3.47	3.46	3.46	331	331	330
Ar–Gr-2%	3.56	4.27	4.45	334	336	337
Ar–Gr-4%	4.08	4.75	6.27	336	344	348
Ar–Gr-6%	4.26	5.28	7.84	338	355	358
Ar–Gr-8%	4.81	6.30	8.28	342	380	389

The effect of temperature on the storage modulus (*E*′) for the two types of composites *i.e.*, ArPrSiGr and ArPhSiGr with different amounts of SiGr is shown in Fig. 5. Starting at 50 °C, the measured modulus of the neat aramid is 3.46 GPa. On addition of SiGr it increases in both types of composite systems. The maximum value in the glassy region for the ArPhSiGr system was 8.28 GPa whereas it was 6.30 GPa for the ArPrSiGr system. These values decrease continually as we enter from glassy to the rubbery region for both types of systems. In the cases of the prepared composites, these values remain higher in comparison to the neat matrix. This can be attributed to the interfacial interaction that facilitated the transfer of the stress from the matrix to the silanized graphene. The primary or the secondary bond interactions between the SiGr and the aramid matrix has clearly participated in providing the load-bearing mechanism and that is responsible for higher modulus in case of composites. ArPhSiGr provided higher values because of rigid phenyl group present in APhTMS than the flexible propyl group present in APrTES. The presence of free electrons and π–π secondary bond interactions due to phenyl groups can be considered as another factor for the higher interphase interaction. The modulus values after initial decrease were found to rise again around 410 °C possibly by the chain alignments due to the cyclic stress as the analysis was carried out in the tensile mode. The drop in the *E*′ values above 480 °C is due to a softening of the polymer.

A comparison in the modulus values for both types of hybrids (Fig. S3†) using amino-silane as coupling with those where pristine graphene was used.^45^ The influence of chemical bonding created by amino-silane with polymer matrix in the freezing of the segmental motion *i.e.*, local chain movements of the polymer chains has increased the elastic properties in the matrix giving much higher values storage modulus and the *T*_g_ in these hybrids as given in the Table 1.

**Fig. 6:**
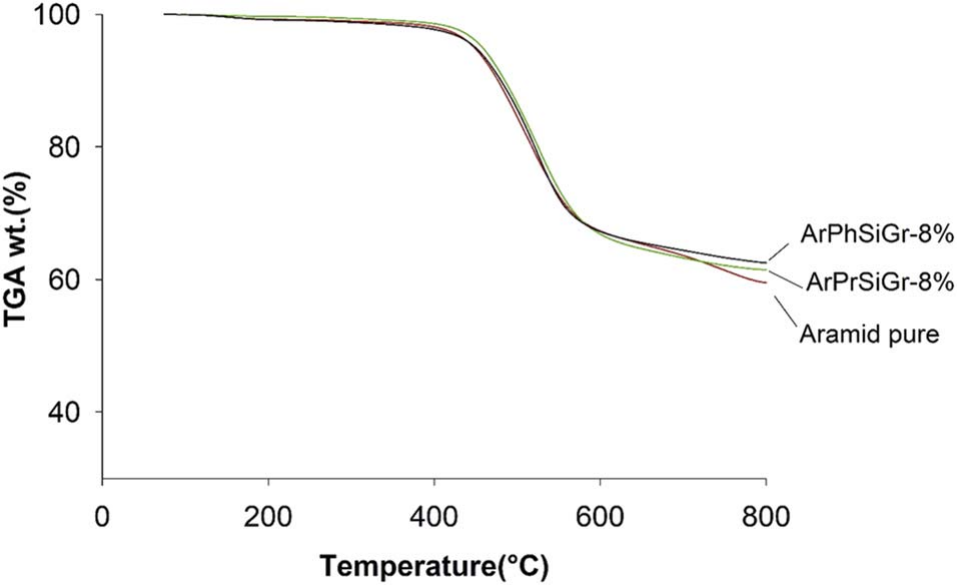
TGA thermograms of aramid pure, ArPhSiGr-8% and ArPrSiGr-8%.

**Fig. 7:**
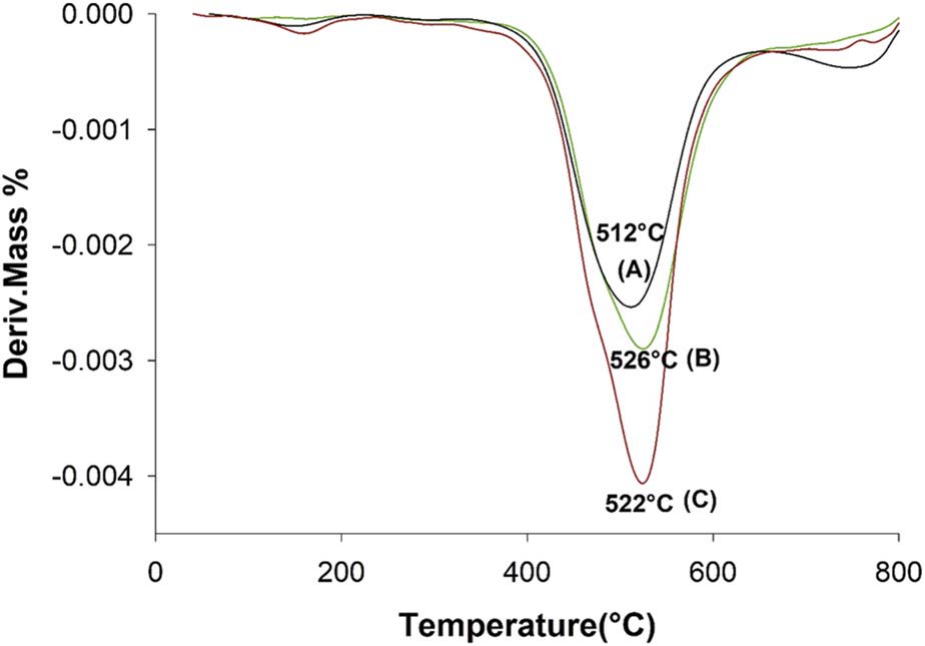
DTGA thermograms of aramid pure (A), ArPhSiGr-8% (B) and ArPrSiGr-8% (C).

The TG curves for the neat aramid, and the composites with 8 wt% SiGr are shown in the Fig. 6. Aramids are considered thermally very stable due to their aromatic character of the backbone. The initial small weight loss observed is due to water absorbed by the polymer film. The degradation of aramid chain starts around 400 °C giving various products, which depend upon the temperature and the environmental conditions, and it includes benzene, amines, HCN and NH_3_ and various carbon products. The weight retained above 800 °C is due to charred material and silica and was the highest in case of ArPhSiGr hybrids. The thermal decomposition temperature of the hybrids was calculated from the first derivative of the TG curves and these graphs are shown in Fig. 7. The linear poly(*p*-phenylene-terephthalamide) chain known as Kevlar© has a thermal decomposition temperature around 528 °C.^56^ To make this type of fiber processable we introduced nearly 20% *meta*-linkages and a thermal decomposition temperature of the matrix was slightly reduced (A) to 512 °C (Fig. 7).

The thermal decomposition temperatures of the graphene hybrids using APhTMS and APrTES shown as (B) and (C) in Fig. 7 were observed at 526 °C and 522 °C respectively and the former have shown a higher increase in comparison to the neat matrix. The oxidation of Gr makes it inherently unstable thermally in comparison to its pristine form, but the silica network created on the both sides of platelets by the sol–gel process improve the stability of SiGr. The network of chemically bonded SiGr in the matrix can protect the aramid matrix to some extent by decreasing the mobility of free radicals produced during the degradation process. Thus, the stability of the composites improves again, and it was almost close to the Kevlar© chain.

## Conclusion

By a careful choice of choosing an organically modified silane, the silica network structure can be produced by the sol–gel process on graphene surface which help to increase the interlayer distance thus disaggregating the graphene platelets and ensuring their uniform distribution in the polymer matrix. With an appropriate choice of functional groups present on silane it can further be utilized to develop chemical bonding between the filler and the matrix. Greater adhesion between the silanized graphene using 3-aminophenyltrimethoxysilane and the aramid chain resulted in a much higher increase in the glass transition temperature and storage modulus, and it was possible to reinforce the polymer with higher contents of filler.

## Conflicts of interest

There are no conflicts to declare.

## Supplementary Material

RA-012-D2RA04797G-s001
