# Supplementary material for: Synthesis and characterization of aramid composites reinforced with silanized graphene platelets
Source: RSC Adv. 2022 Sep 21;12(41):26753–62. doi: 10.1039/d2ra04797g (PMC9490764; doi:10.1039/d2ra04797g)
Supplement: RA-012-D2RA04797G-s001 [file RA-012-D2RA04797G-s001.pdf]

Electronic Supplementary Material (ESI) for RSC Advances.
This journal is © The Royal Society of Chemistry 2022

Support Information

Manuscript ID: RA-ART-08-2022-004797

TITLE: Synthesis and Characterization of Aramid Composites Reinforced with Silanized Graphene Platelets

Abdullah Alhendal*, Jessy Shiju, Mohamed Rashad, Fakhreia Al-Sagheer and Zahoor Ahmad

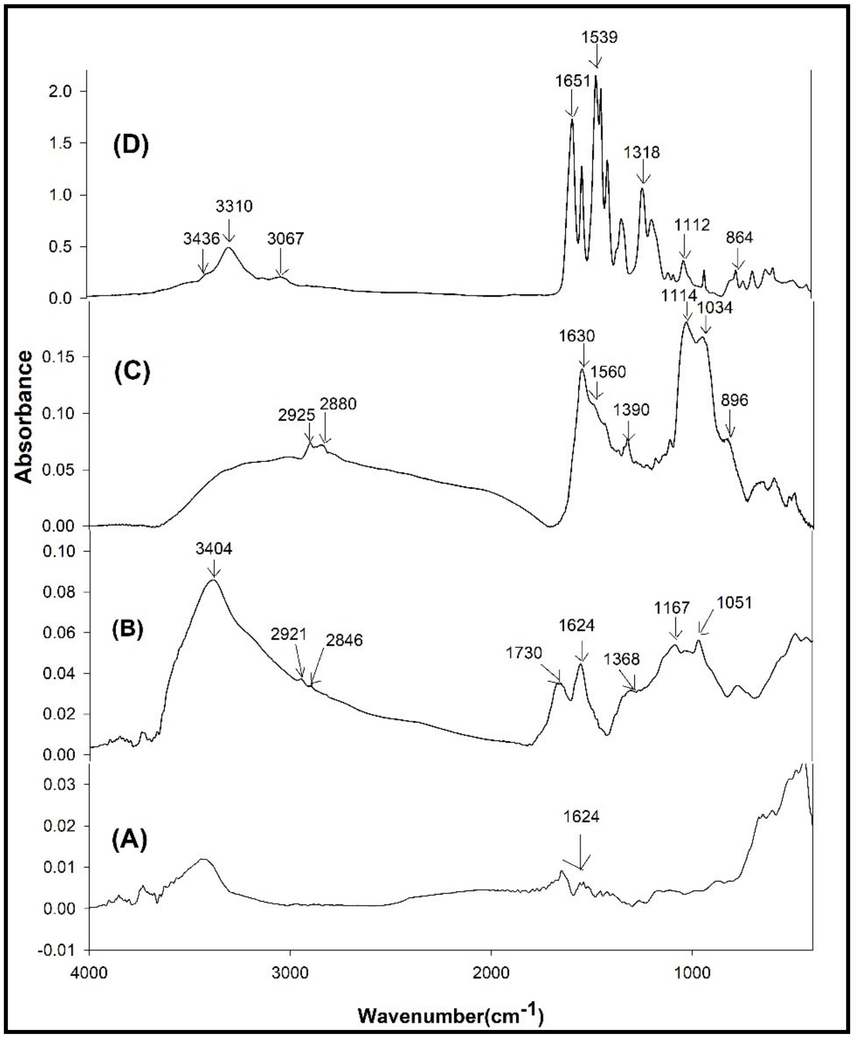

Figure S1 FTIR spectrum of (A): Pristine Gr, (B): GrO, (C): propyl SiGr and (D): Ar ArPrSiGr 8%

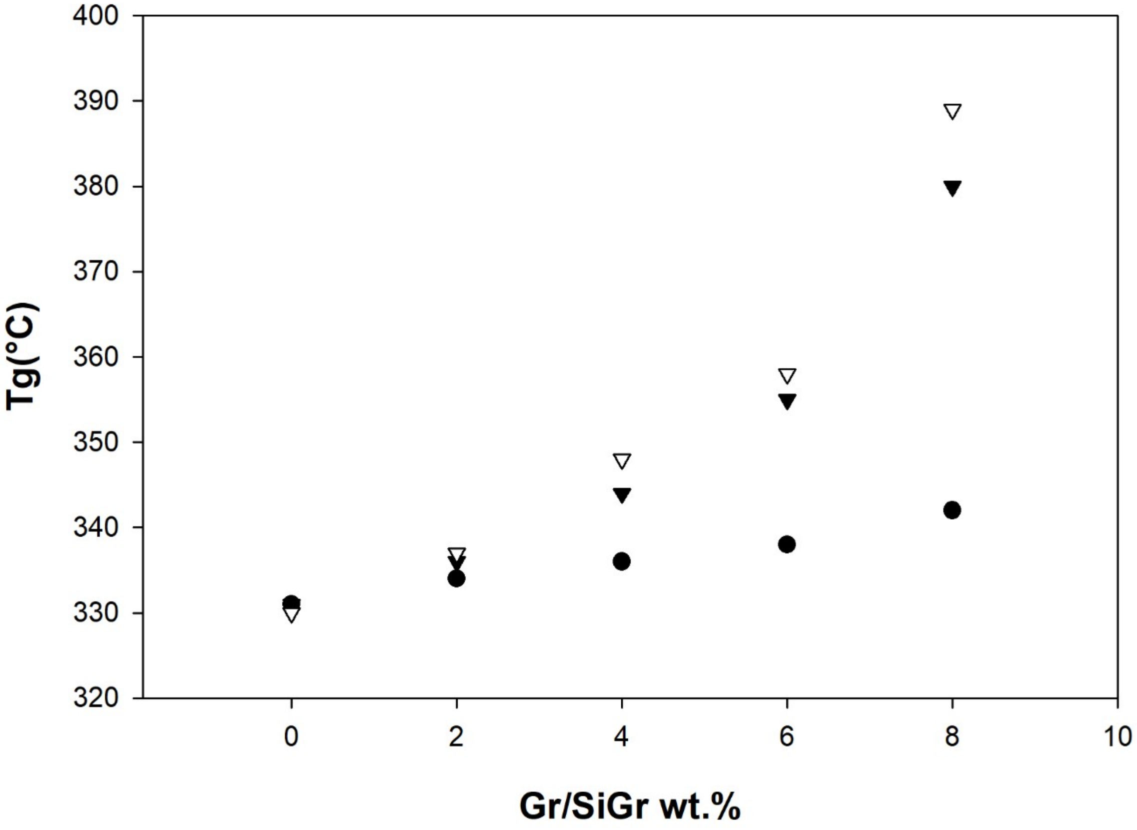

Figure S2. Variation of Tg with filler Wt.% in hybrids : ArGr(●), ArPrSiGr (▼) and ArPhSiGr (▽).

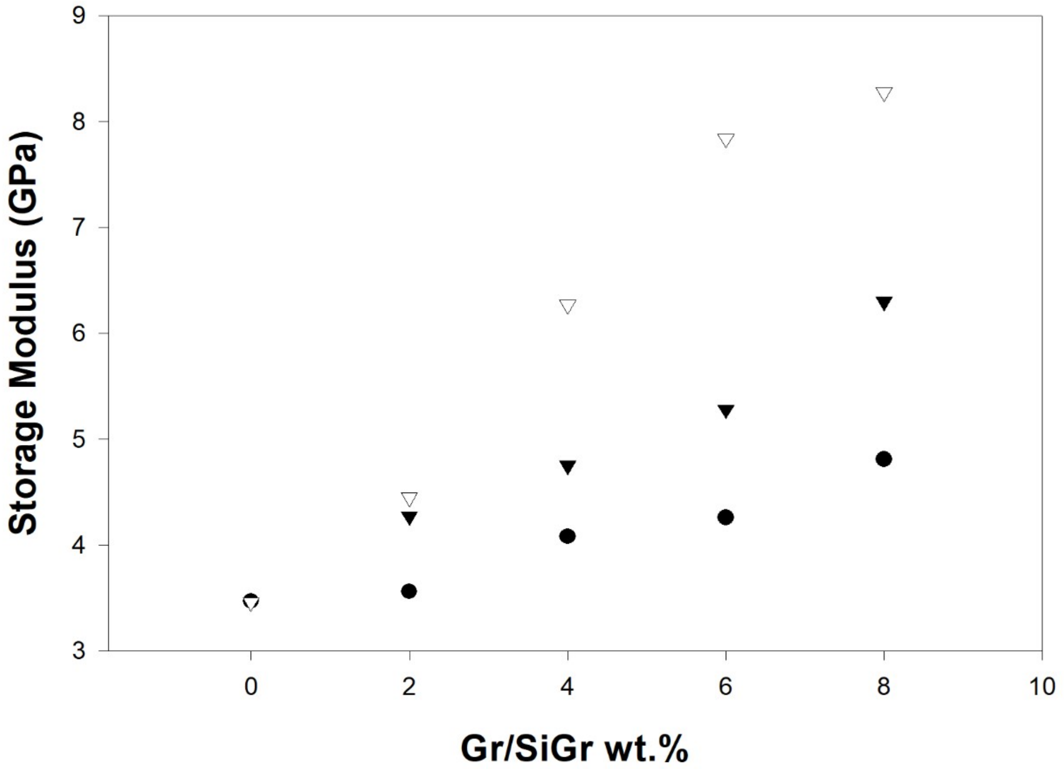

Figure S3. Variation of Storage Modulus with filler Wt.% in hybrids: ArGr(●), ArPrSiGr (▼) and ArPhSiGr (▽)
